# Performance of abiotic stress-inducible synthetic promoters in genetically engineered hybrid poplar (*Populus tremula* × *Populus alba*)

**DOI:** 10.3389/fpls.2022.1011939

**Published:** 2022-10-18

**Authors:** Yongil Yang, Yuanhua Shao, Timothy A. Chaffin, Jun Hyung Lee, Magen R. Poindexter, Amir H. Ahkami, Eduardo Blumwald, C. Neal Stewart

**Affiliations:** ^1^ Center for Agricultural Synthetic Biology, University of Tennessee Institute of Agriculture, Knoxville, TN, United States; ^2^ Department of Plant Sciences, University of Tennessee Institute of Agriculture, Knoxville, TN, United States; ^3^ Biosciences Division, Oak Ridge National Laboratory, Oak Ridge, TN, United States; ^4^ Environmental Molecular Sciences Laboratory (EMSL), Pacific Northwest National Laboratory (PNNL), Richland, WA, United States; ^5^ Department of Plant Sciences, University of California, Davis, Davis, CA, United States

**Keywords:** Synthetic biology, synthetic promoter, osmotic stress, poplar, *cis*-regulatory elements, bioenergy

## Abstract

Abiotic stresses can cause significant damage to plants. For sustainable bioenergy crop production, it is critical to generate resistant crops to such stress. Engineering promoters to control the precise expression of stress resistance genes is a very effective way to address the problem. Here we developed stably transformed *Populus tremula* × *Populus alba* hybrid poplar (INRA 717-1B4) containing one-of-six synthetic drought stress-inducible promoters (SDs; SD9-1, SD9-2, SD9-3, SD13-1, SD18-1, and SD18-3) identified previously by transient transformation assays. We screened green fluorescent protein (GFP) induction in poplar under osmotic stress conditions. Of six transgenic lines containing synthetic promoter, three lines (SD18-1, 9-2, and 9-3) had significant GFP expression in both salt and osmotic stress treatments. Each synthetic promoter employed heptamerized repeats of specific and short *cis*-regulatory elements (7 repeats of 7-8 bases). To verify whether the repeats of longer sequences can improve osmotic stress responsiveness, a transgenic poplar containing the synthetic promoter of the heptamerized entire SD9 motif (20 bases, containing all partial SD9 motifs) was generated and measured for GFP induction under osmotic stress. The heptamerized entire SD9 motif did not result in higher GFP expression than the shorter promoters consisting of heptamerized SD9-1, 9-2, and 9-3 (partial SD9) motifs. This result indicates that shorter synthetic promoters (~50 bp) can be used for versatile control of gene expression in transgenic poplar. These synthetic promoters will be useful tools to engineer stress-resilient bioenergy tree crops in the future.

## Introduction

Abiotic stress causes various damage to agricultural crops and ecosystems, resulting in severe crop yield loss and extra cost for ecosystem recovery (He et al., 2018; El-Sappah et al., 2022). Synthetic biology has tremendous potential to improve trait delivery in food and biofuel crops to resist abiotic stress. For example, recent advances in synthetic biology enabled the installation of genetic circuits and assembly of multigene constructs in transgenic plants for agricultural and pharmaceutical purposes (Lomonossoff and D'Aoust, 2016; Vazquez-Vilar et al., 2018; Andres et al., 2019; Sainsbury, 2020; Yang et al., 2022). While synthetic gene constructs can provide significant versatility, they give rise to a major challenge in the control of gene expression, which is necessary for regulating targeted proteins and the final production of the metabolites of interest (Liu and Stewart, 2015). To address this challenge, many researchers have attempted to control gene expression by generating antisense transcripts against the target transcript or by regulation at the post-transcriptional or post-translational levels (Swiezewski et al., 2009; Martinez de Alba et al., 2013; Van Lijsebettens and Grasser, 2014; Merchante et al., 2017). However, these methods all require sufficient transcription initiation, which is the most efficient target for gene expression controllers. Therefore, the selection of proper promoters is an essential component in both approaches.

In the past, Arabidopsis has been engineered to tolerate abiotic stresses by ectopic expression of the gene encoding dehydration-responsive element binding protein 1A (DREB1A) driven by the 35S Cauliflower mosaic virus (CaMV) promoter (Kasuga et al., 1999). However, the constitutive overexpression of DREB1A was shown to impair plant growth. In contrast to constitutive promoters, specific inducible native promoters or synthetic promoters are useful to express genes of interest in a spatiotemporal fashion. For example, native stress-responsive promoters of *Rab16 A* and *COR14A* were used to induce the synthesis of proteins responding to abscisic acid (ABA), salt, and cold to improve plant growth under stress (RoyChoudhury et al., 2007; Li et al., 2013; Singhal et al., 2016). However, native promoters, because of their plethora of recognizable transcription factor binding sequences (*cis*-elements) associated with stress response and other factors, have complex transcription cues. In order to express stress-responsive genes under a specific stress condition, synthetic promoters made with optimal *cis*-elements can prove useful. One such application of stress-responsive synthetic promoters was in Arabidopsis, in which three synthetic promoters (Ap, Dp, ANDp) were developed from Arabidopsis stress responsive promoters of *responsive to desiccation 29A* (*RD29A*), *RD 29B, dehydration-responsive elements* (*DREs*)*, and ABA responsive elements* (*ABREs*) (Zhang et al., 2022). These promoters were used to induce the gene expressions of cytosolic ABA receptor kinase 1 (CARK1) and regulatory components of ABA receptor 11 (RCAR11), resulting in tolerance against drought stress in Arabidopsis (Ge et al., 2018).

Synthetic promoters have been engineered using *cis*-elements and other transcriptional motifs have been designed and produced for various applications (Ali and Kim, 2019). The positioning and ordering of *cis*-elements in synthetic promoters are important to optimize their function. Recently, Cai et al. (2020) demonstrated an effective methodology for rationally designing short synthetic promoters (Cai et al., 2020). The rational design of a synthetic promoter was conducted with 3-4 copies of a short *cis*-element identified from constitutive promoters relating to pathogen recognition promoter sequences. This short synthetic promoter functioned consistently in transient expression tests using protoplasts of three different plant species, suggesting that short synthetic promoters containing homo- or hetero-repeats of key sequences within active *cis*-elements can enhance targeted gene expression in an orthogonal manner. Another recent study showed the orthogonal regulatory effect of synthetic activators and repressors and their interaction with synthetic promoters using the adopted orthogonal regulatory system of yeast (Belcher et al., 2020). Transient expression assays in *Nicotiana benthamiana* and stable transgenic Arabidopsis were validated sequentially by showing synthetic promoter activation and repression relative to the addition of synthetic promoters.

Owing to the accessibility of genetic information, a reliable transformation system, and abundant experimental work, hybrid poplar species are apt woody biofuel feedstock models (Tuskan et al., 2006; Ma et al., 2019). *Populus* also provides economically valuable bioproducts such as paper, cellulose, wood, and fiber. Poplar has been engineered for improved biofuel production by reducing recalcitrance of lignocellulosic biomass or increasing biomass stock polymers such as cellulose and hemicellulose for conversion to biofuel (Bryant et al., 2020). The overexpression of selected genes or the production of RNA interference fragments of target genes has mainly been controlled by constitutive promoters such as the 35S CaMV promoter or ubiquitin promoter. Additionally, native promoters such as those from the vascular-related NAD transcription factor (VND (SND/NST)) or MYB genes, which serve as regulatory transcription factors for secondary cell wall synthesis and biomass biosynthesis-related enzymes, such as cellulase, have been studied broadly and recently used to develop synthetic promoters in Arabidopsis (Endo et al., 2015; Nakano et al., 2015). Based on native promoter sequence information, small fragments of a secondary cell wall NAC binding element (SNBE) functioned as a synthetic promoter constructed for CRISPR/Cas9 gene editing knock-out of *CINNAMOTYL*-*CoA REDUCTASE 1* (*CCR1*) or stem tissue-specific expression of CCR1 in *ccr1* mutant, resulting in tissue-specific lignin content modification in Arabidopsis (De Meester et al., 2018; Yu et al., 2021). In these studies, the synthetic SNBE promoters were shown to be tissue-specific, showing promise for synthetic promoter motif utility for biomass optimization. Currently however, applicable promoters are limited to biosynthesis processes, not in other areas such as stress response in poplar. Also, most of this work has been performed in herbaceous species, and yet to be confirmed in a woody plant such as poplar.

Our lab recently reported on synthetic drought (SD) inducible promoters designed by computational DNA motif analysis software from the promoter sequences of stress-responsive co-expressed genes in the poplar genome (Yang et al., 2021). The heptamerized 7-8 base repeats of conserved DNA motifs from drought stress-inducible promoters were screened in transient transformation assays using poplar leaf mesophyll protoplasts, agroinfiltration in *N. benthamiana* leaves, and in stably transgenic Arabidopsis. The objective of the present study was to assess the osmotic stress responsiveness of six SD inducible promoters in stably transformed poplar. We also scrutinized the effect of different sequence lengths in a synthetic promoter from a DNA motif with heptamerized repeats of the full-length SD9 motif (20 bases) compared with heptamerized partial sequences (7-8 bases) from the SD9.

## Materials and methods

### Plants

*Populus tremula* × *Populus alba* female INRA 717 1B4 clones (717-1B4) were kindly donated by Dr. Steven Strauss (Oregon State University). Small plants were propagated in solidified rooting media (Yang et al., 2021) in Magenta GA7 boxes (Bioworld, Dublin, OH, USA), and grown in a growth chamber (Percival Scientific, Fontana, WI, USA) with 16/8 light/dark conditions at 23 °C with 150 μmol/m^2^s irradiance. The rooted plants were transferred to ProMix BK25 (Premier Tech, Quakertown, PA, USA) in 1 L pots, and then acclimated for 2 weeks in the same chamber. The propagated plants were grown in a greenhouse at the University of Tennessee, Knoxville, and watered every two days. The potted plants were fertilized with a 14-14-14 nutrient solution (Seed World, Odessa, FL, USA) every three weeks.

*Nicotiana benthamiana* seeds were germinated and grown in ProMix BK25 after 4 days of stratification using sterile water at 4°C in the dark. Plants were grown under the same growth chamber conditions as poplar.

### Generation of binary gene constructs containing inducible synthetic promoters

The binary backbone vectors including a kanamycin resistance gene as a transgenic plant selective marker were adopted from our lab’s published work and used for agroinfiltration testing and transgenic poplar generation (Yang et al., 2021). We screened the same binary vector constructs carrying SD9-1 (a total of 56 bases), 9-2 (49 bases), 9-3 (49 bases), 13-1 (49 bases), 18-1 (56 bases), and 18-3 (49 bases) synthetic promoters in this study. A new synthetic promoter, denoted SD9 (140 bases), was generated from the overlapped core sequences of SD9-1, 9-2, and 9-3, was inserted into the same backbone vector using Golden Gate cloning (Engler and Marillonnet, 2014). The synthesized fragment sequences are listed in **Table S1**. The oligonucleotides for double-strand synthetic promoter fragments were synthesized by Integrated DNA Technologies (IDT, Coralville, IA, USA). All DNA sequences were verified by commercial Sanger sequencing (Psomagen, Rockville, MA, USA).

### Transient agroinfiltration assays in *N. benthamiana* leaves

The binary gene constructs incorporating our synthetic promoters were used for transient agrobacterium infiltration assays. The processes for *Agrobacterium* transformation, agroinfiltration of 1-month-old *N. benthamiana* leaves, osmotic stress treatment, and subsequent fluorescence measurements were performed as previously described (Sparkes et al., 2006; Yang et al., 2021). The GFP fluorescence intensity was measured using a Fluorolog®-3 (HORIBA, Kyoto, JAPAN) fluorescence spectrophotometer using direct scans on intact leaves.

### *Agrobacterium-*mediated poplar transformation and genotyping

*Agrobacterium*-mediated poplar leaf disk transformation was performed with the method established by Song et al. (Song et al., 2006). Leaf disks were collected from two-month-old 717-1B4 poplar plant leaves grown in the Magenta GA7 boxes. Following kanamycin (50 μg/ml) selection regenerated shoots were rooted in rooting medium including activated charcoal (5 g/L) (Kang et al., 2009). The rooted shoots were transplanted into 1 L pots following the protocol described above. After transplanting to soil and growing in the growth chamber for three months, all plants were transplanted into 8 L pots to be grown in the greenhouse.

For genotyping of transgenic poplar, the genomic DNA was extracted from the leaves of each genetic event with a DNeasy® Plant Mini kit (Qiagen, Germantown, MD, USA). PCR was performed by DreamTaq DNA polymerase Master Mixture (Thermo Fisher Scientific) with the reaction sequence as follows: 1 cycle of 95°C for 2 min, 30 cycles repeating 95°C for 30 sec, 57°C for 30 sec, and 72°C for 1 min/1 kb, and 1 cycle of 72°C for 7 min. Two separate reactions were performed, using primers targeting GFP and primers targeting PtUBCc as an internal control for genomic DNA qualification. Bands were separated on 1.2% agarose gels (1 × TAE).

### Ramet propagation of transgenic poplar and osmotic stress treatment

The poplar ramets were propagated from cuttings from the original transgenic plant that grew for at least 6 months in potting mix, and then the ramets were grown for another six to nine months. Fully expanded leaves of propagated ramets were used for stress-responsive testing under high salinity and polyethylene glycol (PEG) treatments. The solutions of 250 mM NaCl (Fisher scientific, Hampton, NH, USA) and 20% PEG 6000 (EMD Millipore Burlington, MA, USA) were prepared with deionized (DI) and autoclaved water. The solutions were directly sprayed onto fully expanded leaves positioned from the fifth to seventh leaf from the main stem apex for 3 days (once daily at 11:00 a.m.). The sprayed leaves were sealed to prevent drying. Leaves in the same position in other ramets were sprayed with DI water as mock control. All stress treatments were performed between March and April of 2021. The stress response was determined by assessing GFP fluorescence using a Fluorolog®-3 (HORIBA, Kyoto, Japan) spectrofluorometer. The corresponding image of multi-channel fluorescence detection was generated with the same leaf using the fluorescence-inducing laser projector (FILP) (Rigoulot et al., 2021). The laser power, filter conditions, and exposure time are described in each figure legend. The image conversion was performed by macro function operated in Image J software (Schneider et al., 2012).

### Statistical analysis

Technical and biological repeat numbers are described in individual figure legends. Statistical analysis of all measurements was performed by the Student’s *t*-test protocol integrated in R (R core team, 2014).

## Results

### Stably transformed poplar with osmotic stress-induced synthetic promoters

To assess our six synthetic promoters (SD9-1, 9-2, 9-3, 13-1, 18-1, and 18-3) *in planta*, we first generated stably transformed poplar each carrying one of the six SD promoters. These six short synthetic promoters were produced from poplar A-domain promoter sequences, which activated downstream GFP expression in transient agroinfiltration assays on *N. benthamiana* leaves under water deficit or high salinity conditions (Yang et al., 2021). Among these synthetic promoters, SD9-2 and SD18-1 reliably induced GFP in transgenic Arabidopsis. However, four other synthetic promoters remained to be tested *in planta*, as well as testing all six promoters in the poplar system.

At least 10 different transgenic events were obtained for each promoter construct *via Agrobacterium-*mediated leaf disk transformation followed by kanamycin selection (data not shown). **Figure S1** summarizes representative images of transformation steps. We confirmed the presence of the *GFP* gene in genomic DNA isolated from seven randomly selected transgenic poplar plants, and three different transgenic events were propagated by cuttings to produce more ramets for the experiments described in the following sections (**Figure S2**).

### Determination of optimal concentrations for salinity and PEG treatments

To determine the optimal conditions for inducing osmotic stress in transgenic poplar, we measured GFP fluorescence on leaves that were sprayed with various concentrations of NaCl and PEG for 3 days. When we first pre-screened by spraying 250 mM NaCl and 30% PEG on several randomly selected transgenic lines, GFP was detected most readily in transgenic poplar lines containing the SD9-2 (*SD9-2::GFP*) and SD18-1 (*SD18-1::GFP*), synthetic promoters, respectively. Therefore, we used two different transgenic events of *SD9-2::GFP* for salt stress experiments and two events of *SD18-1::GFP* for PEG-induced osmotic stress. A transgenic line containing the empty vector was used as a comparator.

**Figure 1:**
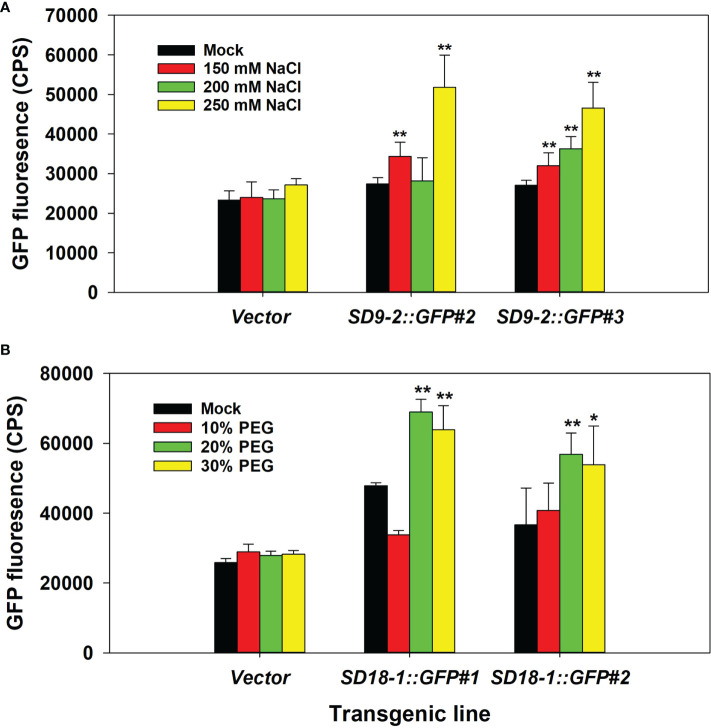
Determination of optimal NaCl and PEG treatment concentrations. The fifth to seventh positioned leaves of six-month-old ramets were used. **(A)** GFP fluorescence measurement after 150, 200, and 250 mM NaCl spray treatment for 3 days in counts per second (CPS). Vector control and two transgenic events of *SD9-2:GFP* as representative transgenic lines were tested. **(B)** GFP fluorescence measurement after 10, 20, and 30% PEG spray treatment for 3 days. Vector control and two transgenic events of *SD18-1:GFP* were used as representative transgenic lines. The bar shows the average of GFP intensity of the abaxial side of treated leaves (n=3). Asterisks denote significant difference of GFP expression between mock control and stress treated leaf *via t*-test (*P < 0.05, **P < 0.01).

In the NaCl treatment test, GFP fluorescence correlated with salt concentrations up to 250 mM (**Figure 1A**). Concentrations of 275 mM and 300 mM resulted in leaf wilting and yellowing, which interfered with the fluorescence measurements (data not shown). Therefore, we selected 250 mM NaCl concentration (~20 ml per spray) to apply to plants every day for 3 days for further salinity treatment tests.

For testing water-deficit conditions, we initially tested withholding water for up to 10 days before measurements. However, leaves were severely shrunken, which affected GFP measurements. Thus, we used PEG chemical treatments to simulate water-deficit conditions that allow proper GFP fluorescence measurement. When leaves were treated with 10, 20, and 30% PEG, the maximum GFP fluorescence was reached in 20% PEG treatments. Therefore, 20% of PEG solutions were used for water-deficit stress measurements in further testing (**Figure 1B**).

### Synthetic promoter activity using the SD9 motif with heptamerized partial *cis*-regulatory sequences (7-8 bases) versus a heptamerized full SD9 motif (20 bases)

**Figure 2:**
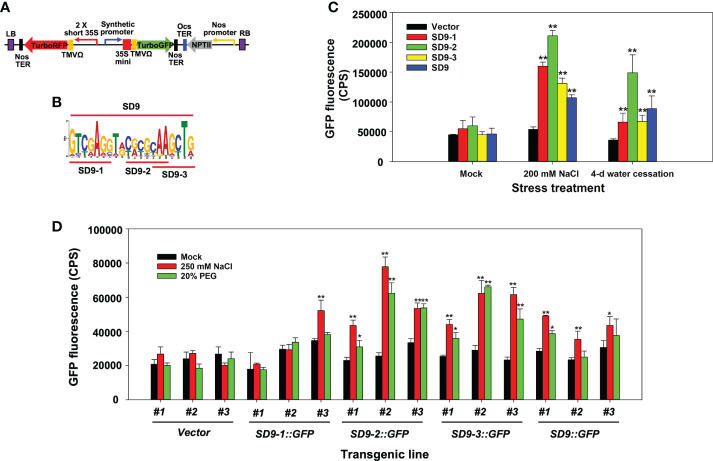
Stress induction of synthetic promoters consisting of heptamerized partial or full sequences from the SD9 motif. **(A)** Binary vector construct containing the synthetic promoter fused to TurboGFP. TurboRFP was driven by a 2 × short 35S constitutive promoter to monitor transformation. Heptamerized partial or full sequences from the SD9 motif (panel **(B)** were inserted into this backbone plasmid. **(B)** LOGO plot of full and partial DNA sequences of SD9. This sequence was heptamerized in working plasmids. **(C)** Transient expression analysis by agroinfiltration on *N. benthamiana* leaves. Each bar represents the average of GFP fluorescence measured by Fluorolog fluorescence spectrophotometry (n=3) in CPS. **(D)** Stress-inducible analysis of synthetic promoters consisting of heptamerized partial or full sequences from the SD9 motif in stable transgenic poplar. The GFP expression in the transgenic poplar leaves each carrying synthetic promoters in high salinity or drought-like conditions. For both tests, the leaves of six-month-old propagated clones were sprayed with 250 mM NaCl or 20% PEG for 3 days. Mock control was sprayed with DI water for the same period. The bar shows the average of GFP intensity of the abaxial side of treated leaves (n=3). In panel c and d, error bar represents standard deviation. The statistical significance of each value against vector control was determined by *t*-test (*P < 0.05, **P < 0.01).

To identify if the synthetic promoter activity is dependent on core sequence length, we assessed whether a synthetic promoter formation with repeats of the full SD9 motif sequence (20 bases) functions more effectively than the heptamerized partial sequences (7-8 bases; SD9-1, SD9-2, and SD9-3). For this purpose, we generated a synthetic promoter of the entire SD9 motif (20 bases) and cloned it into a binary vector (**Figures 2A, B**).

Transient *Agrobacterium* infiltration experiments on leaves of *N. benthamiana* were performed to visualize the synthetic promoter activity in leaves under high salinity and severe water-deficit stress. Separate treatments of watering with 200 mM NaCl and water cessation were used to generate osmotic stress conditions after agroinfiltration (Yang et al., 2021). *SD9::GFP* had consistently lower GFP expression compared to the partial sequence repeats while *SD9-2::GFP* induced higher promoter activity and GFP gene expression than the others under both high salt and water-deficit conditions (**Figure 2C**). Interestingly, all synthetic promoters were more induced by salt than water deficit stress.

These results were confirmed in stably transformed poplar plants with the same gene constructs used for transient agroinfiltration assays. Three different transgenic events were established for each gene construct transformation (**Figure 2D**). Leaves of six-month-old propagated transgenic poplar ramets were sprayed separately with 250 mM NaCl or 20% PEG for 3 days. Leaves of *SD9::GFP*, *SD9-2::GFP*, and *SD9-3::GFP* transgenic poplars showed up to 2-fold higher GFP fluorescence than mock treatments after exposure to 250 mM NaCl (**Figure 2D**). These synthetic promoters also induced GFP expression in 20% PEG treatment, but the fluorescence intensity was relatively lower than that of salt treated leaves (**Figure 2D**). In line with our observations in the transient agroinfiltration tests, the heptamerized SD9-2 and 9-3 synthetic promoters induced around 1.5-fold higher than that of the complete SD9 in the 250 mM NaCl treatment. In contrast with the agroinfiltration tests, the heptamerized SD9-1 did not induce GFP expression for either stress condition.

To summarize, three transgenic poplar lines containing the heptamerized SD9-2, 9-3, and the entire SD9 synthetic promoter showed responses to NaCl and PEG. However, the synthetic promoters constructed with heptamerized partial sequences of SD9 (SD9-2 and 9-3) showed higher promoter activity than the heptamerized SD9 full sequence (SD9) in high salinity and PEG treatments.

### Osmotic stress responses of stably transformed poplar carrying synthetic promoters derived from the SD9 motif

**Figure 3:**
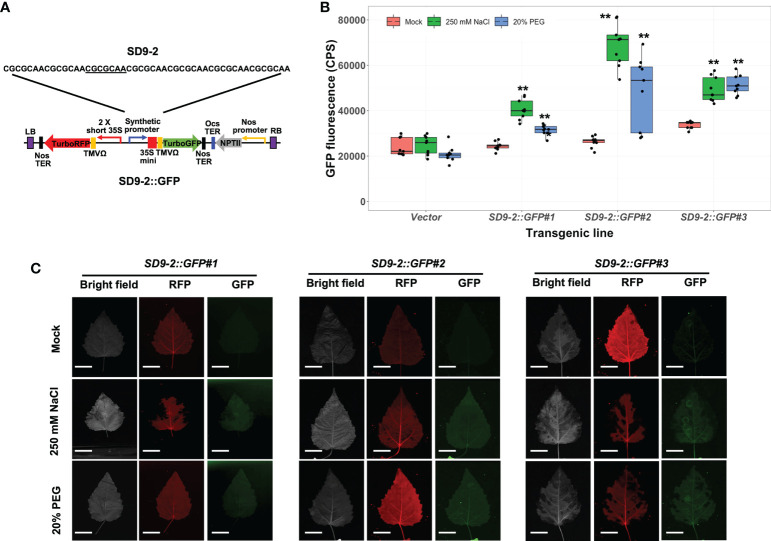
Osmotic stress induction of GFP fluorescence in transgenic poplar containing SD9-2 synthetic promoter (*SD9-2::GFP*). Three different transgenic poplar lines were propagated by cutting and grown in the greenhouse. **(A)** SD9-2::GFP gene construct for generating transgenic poplar. Heptamerized 7 base sequence (basic sequence of CGCGCAA; SD9-2) was inserted into this binary vector. **(B)** GFP fluorescence in transgenic poplar carrying SD9-2 synthetic promoter under osmotic stress treatments. Six- to nine-month-old transgenic poplar lines were treated with 250 mM NaCl or 20% PEG. NaCl and PEG solutions were sprayed for 3 days on the leaves selected between fifth and seventh positions from the apex. Water was sprayed for mock treatment (mock) following the same guidelines. CPS values of GFP fluorescence were measured by Fluorolog fluorescence spectrophotometry at emission wavelength of 502 nm under a fixed excitation wavelength of 482 nm. Triplicate experiments were performed with three different clones (n=9). Asterisks denote significant difference of GFP expression between mock control and stress treated leaf *via t*-test (*P < 0.05, **P < 0.01). **(C)** Visualizations of induced RFP and GFP fluorescence detected by FILP, using the same stress-treated leaves. RFP was used as an internal control to monitor for transformation. Exposure time for images was 100 ms. Scale bars represent 2 cm of length at a detection distance of 3 m.

To verify the osmotic stress responsiveness of SD9-1, 9-2, and 9-3 synthetic promoters in stable transgenic plants, we measured GFP fluorescence in transformed poplar after 250 mM or 20% PEG treatments for 3 days. Among three SD9-derived synthetic promoters, the transgenic poplar containing SD9-1 (*SD9-1::GFP*) did not display *GFP* induction despite previous transient transformation tests that showed *GFP* induction under osmotic stress (**Figure S3**; Yang et al., 2021). However, all three transgenic event lines carrying the SD9-2 synthetic promoter (*SD9-2::GFP*) had significantly induced higher GFP expression (1.5 to 2.6-fold) in 250 mM NaCl treatment than mock control (**Figure 3B**). *SD9-2::GFP* displayed 1.2 ~ 1.8-fold GFP fluorescence increase in response to the 20% PEG treatments (**Figure 3B**). Multi-channel fluorescence scanning images of salt or PEG treated leaves using FILP showed clear and consistent activation of GFP fluorescence (**Figure 3C**). RFP fluorescence, an internal control for monitoring correct transformation, was detected in all mock control and treated leaves, confirming that the gene constructs were transformed stably into transgenic poplar (**Figure 3C**). The binary vector construct for poplar transformation is shown in **Figure 3A**.

**Figure 4:**
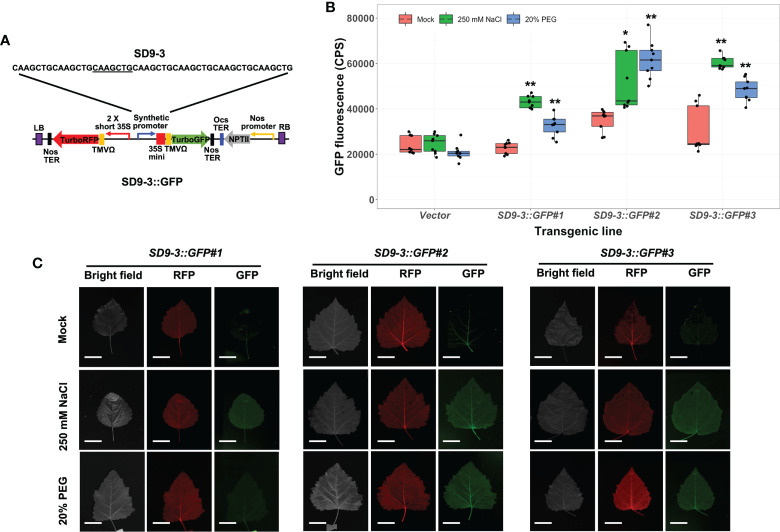
Osmotic stress induction of GFP fluorescence in transgenic poplar carrying SD9-3 synthetic promoter (*SD9-3::GFP*). Three different transgenic poplar lines were propagated by cutting and grown in the greenhouse. **(A)** SD9-3::GPF gene construct for generating transgenic poplar. Heptamerized 7 bases (CAAGCTG; SD9-3) was inserted into this binary vector fused to GFP. **(B)** GFP fluorescence in transgenic poplars containing SD9-3 synthetic promoter under osmotic stress treatments. Six- to nine-month-old transgenic poplar lines were treated with 250 mM NaCl, or 20% PEG. NaCl and PEG solutions were sprayed for 3 days on the leaves selected between fifth and seventh positioned from apex. Water was sprayed for mock treatment (mock). CPS value of GFP fluorescence was measured at emission wavelength of 502 nm under a fixed excitation wavelength of 482 nm measured by Fluorolog fluorescence spectrophotometry in CPS. Triplicate experiments were performed with three clones (n=9). Asterisks denote significant difference of GFP expression between mock control and stress treated leaf *via t*-test (*P < 0.05, **P < 0.01). **(C)** Visualization of induced RFP and GFP fluorescence detected by FILP, using the same stress-treated leaves. RFP was used as an internal control to confirm gene construct transformation. Exposure time for images was 100 ms. Scale bars represent 2 cm of length at a detection distance of 3 m.

The transgenic poplar lines carrying the SD9-3 synthetic promoter (*SD9-3::GFP*) had GFP fluorescence ranging from 1.5 to 2.0-fold higher in 250 mM NaCl-treated leaves compared to the mock control (**Figure 4B**). In the 20% PEG treatments, the GFP fluorescence ranged from 1.4- to 1.8-fold higher (**Figure 4B**). The fluorescence images taken by FILP were consistent with the fluorolog-measured GFP fluorescence (**Figure 4C**). The GFP fluorescence intensity for SD9-3 was similar to the range recorded for SD9-2. In summary, of the three SD9-derived synthetic promoters, SD9-2 and SD9-3 demonstrated similarly high responses to high salinity and PEG, while SD9-1 did not respond in transgenic poplar lines.

### Osmotic stress responses of stably transformed poplar carrying synthetic promoters derived from the SD13 and SD18 motifs

**Figure 5:**
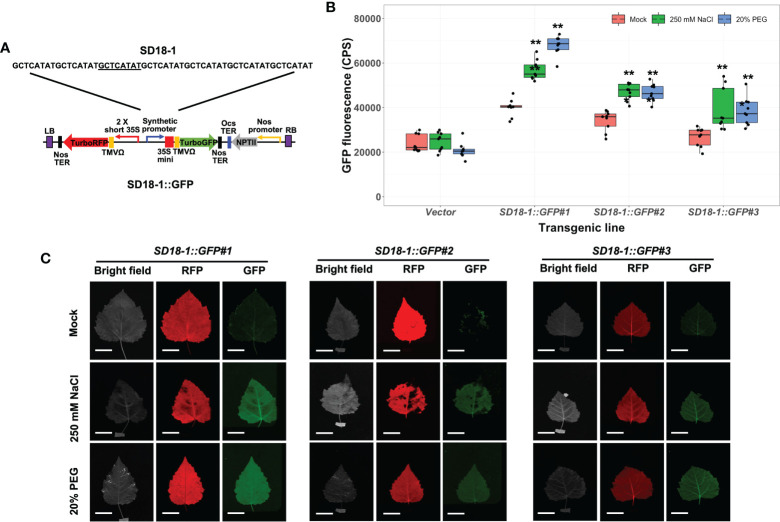
Stress induction of GFP fluorescence in transgenic poplar harboring SD18-1 synthetic promoter (*SD18-1::GFP*) by osmotic stress. Three different transgenic poplar lines were propagated by cutting and grown in a greenhouse. **(A)** SD18-1::GFP gene construct for generating transgenic poplar. Heptamerized 8-base sequence (GCTCATAT; SD18-1) was inserted into binary vector fused to GFP. **(B)** GFP fluorescence in transgenic poplars harboring SD18-1 synthetic promoter under osmotic stress treatments. Six- to nine-month-old transgenic poplar lines were treated with 250 mM NaCl or 20% PEG. NaCl and PEG solutions were sprayed for 3 days on the leaves selected between fifth and seventh positions from apex. Water was sprayed for mock treatment following the same guidelines (mock). CPS of GFP fluorescence was measured at emission wavelength of 502 nm under a fixed excitation wavelength of 482 nm measured by Fluorolog fluorescence spectrophotometry. Triplicate experiments were performed with three different clones (n=9). Asterisks denote significant difference of GFP induction between mock control and stress treated leaf *via t*-test (*P < 0.05, **P < 0.01). **(C)** The image of induced RFP and GFP fluorescence detected by FILP, using the same stress-treated leaves. RFP was used as an internal control to confirm gene construct transformation. Exposure time for images was 100 ms. Scale bars represent 2 cm of length at a detection distance of 3 m.

We verified osmotic stress sensitivity of other SD promoters in stably transformed poplar plants after 250 mM NaCl and 20% PEG treatments for 3 days. Two synthetic promoters from the SD13 motif (SD13-1 and 13-2) and three synthetic promoters with the SD18 motif (SD18-1, 18-2, and 18-3), which presented together with SD9 in the poplar A-domain, were tested (Yang et al., 2021). Of these GFP fused binary vectors, the SD13-1, 18-1, and 18-3 synthetic promoters were significantly activated by high salinity and water-deficit stresses. However, present transgenic poplar plants containing SD13-1 (*SD13-1::GFP*) and SD18-3 synthetic promoters (*SD18-3::GFP*) did not have increased GFP fluorescence when treated in the 250 mM NaCl or 20% PEG treatment (**Figure S3**). Of these three synthetic promoters, only transgenic poplar with the SD18-1 synthetic promoter (*SD18-1::GFP*) had significantly induced GFP expression in the range from 1.4- to 1.5- fold and 1.5- to 1.7-fold higher GFP fluorescence in NaCl and PEG treatments, respectively (**Figure 5B**). As we observed in *SD9-2::GFP* and *SD9-3::GFP*, the FILP images were consistent with measured GFP fluorescence (**Figure 5C**). However, the GFP fluorescence value was 0.7-fold lower (average of all GFP fluorescence in three transgenic events) than that of *SD9-2::GFP* and *SD9-3::GFP*.

### Summary of transient and stable transformation tests of SD synthetic promoters

In summary, we performed transient transformation using poplar leaf mesophyll protoplasts and agroinfiltration of *N. benthamiana* leaves, as well as stable transformation of poplar using the same binary vectors each carrying one of our synthetic promoters. Through GFP fluorescence assays, we identified the stress responsiveness of poplar osmotic stress-induced synthetic promoters. The results for these assays are summarized in **Table 1**. Out of eight SD-series synthetic promoters, six synthetic promoters including SD9-1, 9-2, 9-3, 13-1, 18-1, and 18-3 activated downstream GFP expression in transient transformation tests under osmotic stress conditions (Yang et al., 2021). Three SD synthetic promoters including SD9-2, 9-3, and 18-1 also consistently induced GFP expression in stable transgenic poplars (**Figures 3**–**5**). These three synthetic promoters responded to both high salinity and PEG treatments. The osmotic stress responsiveness of synthetic promoters was reliably screened in both transiently and stably transformed plant cells although a small number of biases still existed between both transformations. Three SDs were confirmed as osmotic stress-responsible synthetic promoters through all tests.

**Table 1:** Summary of GFP induction of SD synthetic promoters in transient and stably transformed plants under osmotic stress conditions. .

Synthetic promoter	Poplar mesophyll protoplast	Agroinfiltration in *N. benthamiana* leaves			Stably transformed transgenic poplar	
	0.5 M mannitol	3-day 200 mM NaCl	3-day 100 mM mannitol	4-day water cessation	3-day 250 mM NaCl	3-day 20% PEG
SD9-1	+	+	–	–	+	–
SD9-2	++	++	++	++	++	++
SD9-3	++	++	++	+	++	++
SD13-1	++	–	–	++	–	–
SD13-2	+	n.a.			n.a.	
SD18-1	++	–	++	++	++	++
SD18-2	–	n.a.			n.a.	
SD18-3	++	–	++	+	–	–

This table summarizes previously published results of transient experiments performed by our lab (Yang et al. 2021) as well as present results in stably transformed transgenic poplar using the same binary plasmids. Significant GFP increase is denoted by + mark (+: P < 0.05; ++: P < 0.01; -: not detected; n.a.: not applied). Significance was determined by comparing to GFP intensity in vector plasmid transformed protoplast for protoplast transformation test. The others were compared against mock control.

## Discussion

Modern synthetic biology techniques for transgenic plant generation have been regarded as powerful tools for revolutionizing agriculture. Promoter modification with native or synthetic promoters is a powerful approach for tuning targeted gene expression in transgenic plants. To control gene expression at the optimal timing and with the appropriate transcript abundance, it is necessary to design versatile synthetic promoters, which considers optimal sequence selection, copy number and spacing optimization, and proper orientation and order of components within the synthetic promoter. Most of all, it is important that researchers select constituent components that maximize the activity of promoter constructs including three important elements: a) the core promoter, b) appropriate TF binding site(s) in the proximal promoter, and c) other additive elements such as enhancers or insulators (repressors) as needed for the application of interest (Dey et al., 2015). To date, native and synthetic promoters for this purpose have been mostly reported in a few species (Dey et al., 2015; Liu and Stewart, 2016; Ali and Kim, 2019).

We previously reported poplar osmotic stress-responsive synthetic promoters that were based on native promoter sequences from *Populus*. The CaMV 35S core and translation leader 5’ untranslated region (UTR) sequences were fused to a *GFP* reporter gene, and GFP activity was assayed by transient screening using poplar protoplasts and agroinfiltration (Yang et al., 2021). The present study confirmed the positive activity of these synthetic promoters in stably transformed poplar plants under osmotic stress (**Figures 3**–**5**, and **Table 1**). The synthetic promoters are composed of synthetic heptamer repeats fused with the -46 35S minimal core promoter, which includes a TATA boxn, and a TMV Ω 5’ UTR leader sequence to maximize translational efficiency. Recent studies using STARR-seq analysis showed that the most robust core promoters have the TATA box at the -30 to -40 bp location from the transcriptional starting site (TSS) (Jores et al., 2020; Jores et al., 2021). They found that core promoters, which include the TATA box, activated downstream gene expression in a species-specific manner. In addition, they also identified peak promoter activity when the core promoter worked in tandem with other transcription initiation factors such as an initiator and pyrimidine-rich promoter (TC motif or Y-patch) (Jores et al., 2021). Several DNA sequence studies have used high throughput techniques to study core promoter sequences from yeast, *Drosophila melanogaster*, and human cells (Lubliner et al., 2015; Arnold et al., 2017; van Arensbergen et al., 2017; Weingarten-Gabbay et al., 2019; de Boer et al., 2020; Kotopka and Smolke, 2020). These studies revealed that the core promoter elements and TF-binding sites in the region close to the TSS cooperate to mediate the gene transcription. Other *cis*-elements such as enhancers and insulators can be added distal to the promoter region for the regulation of synthetic promoter activity (Lenhard et al., 2012; Beagrie and Pombo, 2016; Haberle and Stark, 2018). However, these findings had limited applications in plant systems because core promoter elements including the TATA box and the initiator (Ins) elements were identified by computational analysis without reliable confirmation *in planta* (Yamamoto et al., 2007; Morton et al., 2014; Srivastava et al., 2014; Jores et al., 2020). It was recently reported, using massively parallel reporter assays in tobacco leaves and maize protoplasts, that the core promoter elements including the TATA box as well as promoter GC content and promoter proximal transcription factor binding sites affect the strength of most promoters in the genes of Arabidopsis, maize, and sorghum (Jores et al., 2021). The study demonstrated that promoter-proximal *cis*-regulatory elements work in tandem with the core promoter sequence to determine expression strength in tobacco leaves and maize protoplasts (Jores et al., 2021). In addition, another study screened a 37-promoter library comprised of fourteen common plant active promoters, seven 5’ UTRs, and eleven 3’ UTRs in transfected *N. benthamiana* leaves, canopies, and protoplasts. This study showed that 3’ UTR components are also important for maximizing promoter activity, in addition to upstream regulatory elements (Pfotenhauer et al., 2022). Therefore, understanding the cooperation of entire promoter compositions including the core elements, proximal *cis*-elements close to the TSS, other factors such as enhancers and insulators (or repressors), and 3’ UTR of transcription terminator is crucial for the design of versatile synthetic promoters. Although our present gene construct demonstrated coordination between each component, it is yet to be determined if further optimization is possible for gene expression in poplar. In order to generate the optimized synthetic promoter, it will be necessary to test other features such as replacement of the current virus core promoter with an intact poplar core promoter or linkage with other candidate *cis*-elements.

Another important factor for consideration is the ability of proximal *cis*-elements of the core promoter fragment to modify and boost promoter activity (Cai et al., 2020; Yang et al., 2021). In the present study as well as our previously published results, we tested many unknown *cis*-elements from the poplar A-domain as well as salt stress-responsive promoters found in the poplar genome (Yang et al., 2021). Using heptamerized 7-20 bp core sequences, we successfully showed *GFP* induction in poplar leaf protoplasts, transiently agroinfiltrated *N. benthamiana* leaves, and stably-transformed transgenic Arabidopsis and poplar plants after osmotic stress treatments (**Figures 2**–**5** and Yang et al., 2021). Our results showed that the promoter activity of the heptamerized entire SD9 motif including SD9-1, 9-2, and 9-3, resulted in lower *GFP* induction, compared to that in separate SD9-2 and 9-3 short DNA motifs, despite those sequences being contained within SD9 (**Figure 2**). The higher synthetic promoter activity using 7 copies of basic *cis*-elements supports the observation that a 3-4 repeat combination of different *cis*-elements had stronger promoter activity than a single copy of a short *cis*-element (Cai et al., 2020). Interestingly, our results showed that the synthetic promoter containing 7 repeats of a short sequence induced stronger downstream gene expression than that of the same repeats of a longer sequence, indicating that promoter activity is not associated with the length of its sequence per se. These results demonstrate the importance of understanding individual *cis*-element features and their interactions to rationally design functional synthetic promoters.

Cooperation or repression effects *via trans* factors, such as the transcription factor protein (TF) or its complex, may also be important considerations for the optimization of synthetic promoter activity. There are many crucial transcription regulators that maintain typical cellular events for cell development and determination, tissue homeostasis, response to environmental stimuli, and disease processes (Maurano et al., 2012; Andersson et al., 2014; Liu and Stewart, 2015).

Promoters have been classically defined as an activating regulatory element together with an enhancer that interacts with the main promoter to amplify transcription dose (Shlyueva et al., 2014; Beagrie and Pombo, 2016; Haberle and Stark, 2018). With the advancement of modern genomic techniques, it has been discovered that the locations of the enhancer and promoter relative to the TSS, as well as the interaction between these factors, are important factors for transcription regulation (Andersson and Sandelin, 2020). Promoters serve a critical role in establishing baseline transcriptional capacity through the recruitment of proteins, including transcription factors. Because of the complexity of the TF complex and its simultaneous role as an activator and repressor, it is still difficult to explain how and where TFs and their cognizant binding of *cis*-elements interact for synthetic promoter activity. As high-throughput sequencing continues to advance, it will become easier to elucidate the tissue-specific or cell-specific expression of the TF complex through techniques using single-cell analysis (Dorrity et al., 2021; Marand et al., 2021; Zhang et al., 2021), allowing the use of TF-modulation of synthetic promoters in plant systems (Belcher et al., 2020). The synthetic neurospora-based ‘Q-system’ has previously been applied to enhance synthetic promoters to tune signal specificity and sensitivity in Arabidopsis and potato (Persad et al., 2020; Persad-Russell et al., 2022). Modifying or identifying a similar orthogonal system could help to improve synthetic promoter activity in other species such as poplar.

To date, the functional identification of distal regulators such as enhancers and silencers in the poplar genome has been scarce. Further studies are needed for the identification of enhancers to maximize synthetic promoter activity. In addition, assessing tissue- or cell-specific factors from various promoters can contribute to the generation of multiple functional synthetic promoters based on our current design. To our knowledge, the present study is the first to confirm osmotic stress-responsive synthetic promoter designs in a transgenic woody plant. We expect to expand upon this success and develop more useful techniques for industrial applications, contributing to the use of poplar and other woody plant species as a sustainable feedstock for biofuel applications in marginal areas with salty soil and subject to drought conditions. Utilizing new promoter sequences as outlined in this study is the first step in developing stronger abiotic stress-responsive synthetic promoters for long-lived trees.

## Conclusion

To construct valuable stress-responsive synthetic promoters for use in woody plants, we characterized transgenic poplar lines containing the SD promoters consisting of 7 copies of short core sequences. Three synthetic promoters responded positively against osmotic stresses in transgenic poplar. In addition, higher GFP induction by short SDs (~50 bases) containing 7 copies of shorter core sequences than the one with longer (~140 bases) sequences implies that longer sequences are not necessarily required for optimal gene expression. The present study confirmed the stress responsiveness of SDs in transgenic poplar, activities of which were predicted by transient transformation assay following the promoter synthesis from computational analysis.

The present results show a good process from core sequence selection to promoter verification of developing synthetic promoters in woody plants. However, further work is needed to develop optimized synthetic promoters for activation under specific abiotic stresses. Rational design using optimal positioning and ordering of core sequences is necessary. Furthermore, effective components such as enhancers and orthogonal factors are required for the amplification of current synthetic promoters’ activities. In the future, the identified synthetic promoters will be fused with stress-responsive genes and stress-related hormone genes, which can enhance stress tolerance and rapid recovery of cell damage in transgenic wood.

## Data availability statement

The original contributions presented in the study are included in the article/**Supplementary Material**. Further inquiries can be directed to the corresponding author.

## Author contributions

YY contributed to designing and performing the experiments, analyzing the data, and writing the manuscript. YS, TC, JL, and MP generated transgenic poplar and cared for plants. YS and TC additionally helped with collecting fluorescence measurement data. AA, EB, and CNS conceived of the study and its design and coordination and assisted with interpretation of results and versions of the manuscript. All authors read and approved the final manuscript.

## Funding

This work was supported by funding from the Biological and Environmental Research in the U.S. Department of Energy Office of Science (DE-SC0018347).

## Acknowledgments

We are especially appreciative of our collaboration with Steve DiFazio and his ideas and discussions with him. We also appreciate the support of technical staff and students that contributed to the work in our laboratories.

## Conflict of interest

CNS is an inventor on synthetic promoter and biotechnology intellectual property that is assigned to the University of Tennessee Research Foundation.

The remaining authors declare that the research was conducted in the absence of any commercial or financial relationships that could be construed as a potential conflict of interest.

## Publisher’s note

All claims expressed in this article are solely those of the authors and do not necessarily represent those of their affiliated organizations, or those of the publisher, the editors and the reviewers. Any product that may be evaluated in this article, or claim that may be made by its manufacturer, is not guaranteed or endorsed by the publisher.
