# Supplementary material for: Performance of abiotic stress-inducible synthetic promoters in genetically engineered hybrid poplar (Populus tremula × Populus alba)
Source: Front Plant Sci. 2022 Oct 18;13:1011939. doi: 10.3389/fpls.2022.1011939 (PMC9623294; doi:10.3389/fpls.2022.1011939)
Supplement: Supplementary file 1 [file DataSheet_1.docx]

Yang supplement


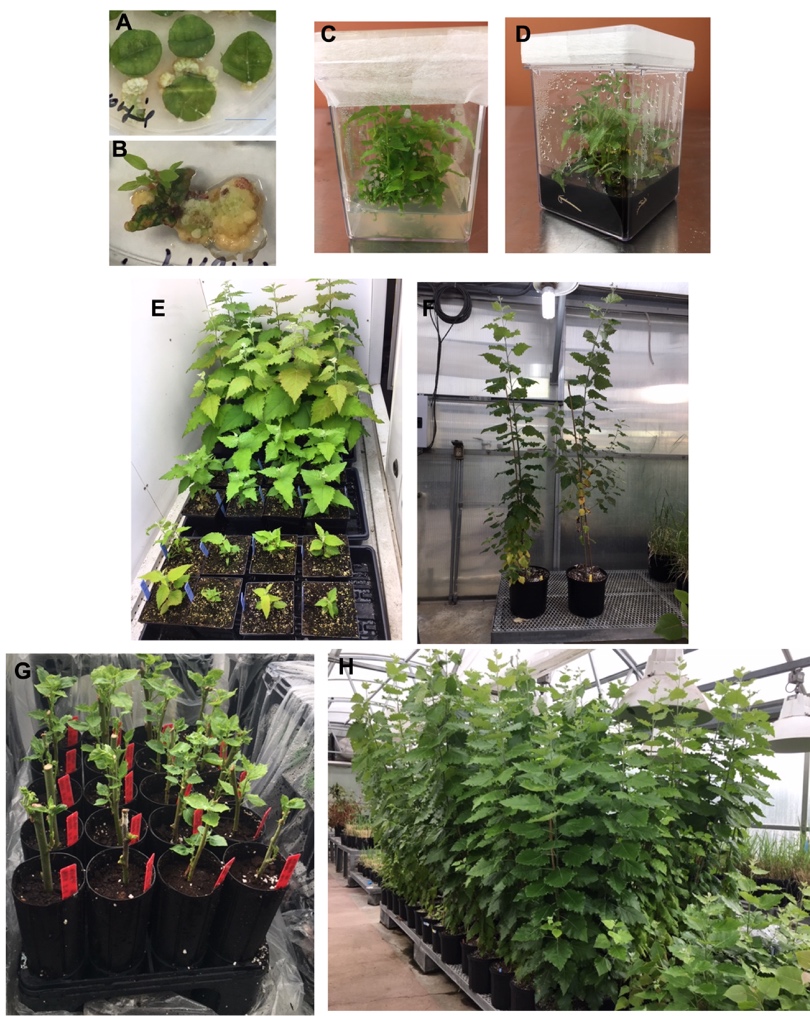


Figure S1. **Representative images of transgenic poplar tissue and plants.**

(A) Transgenic callus induced on leaf disk under kanamycin selection (50 μg/ml). (B) A single transgenic shoot regenerated from callus. (C) Elongated shoots regenerated from transgenic callus. (D) Micropropagation of regenerated whole transgenic poplar plants on rooting media. (E) Rooted plants transplanted in soil and grown in growth chamber. (F) Plants transplanted to large pots for further growth in greenhouse for cutting propagation. (G) Shoots propagated by stem cutting from full grown initial transgenic poplar (panel F) in greenhouse. (H) Propagated transgenic poplar plants used in greenhouse experiments.


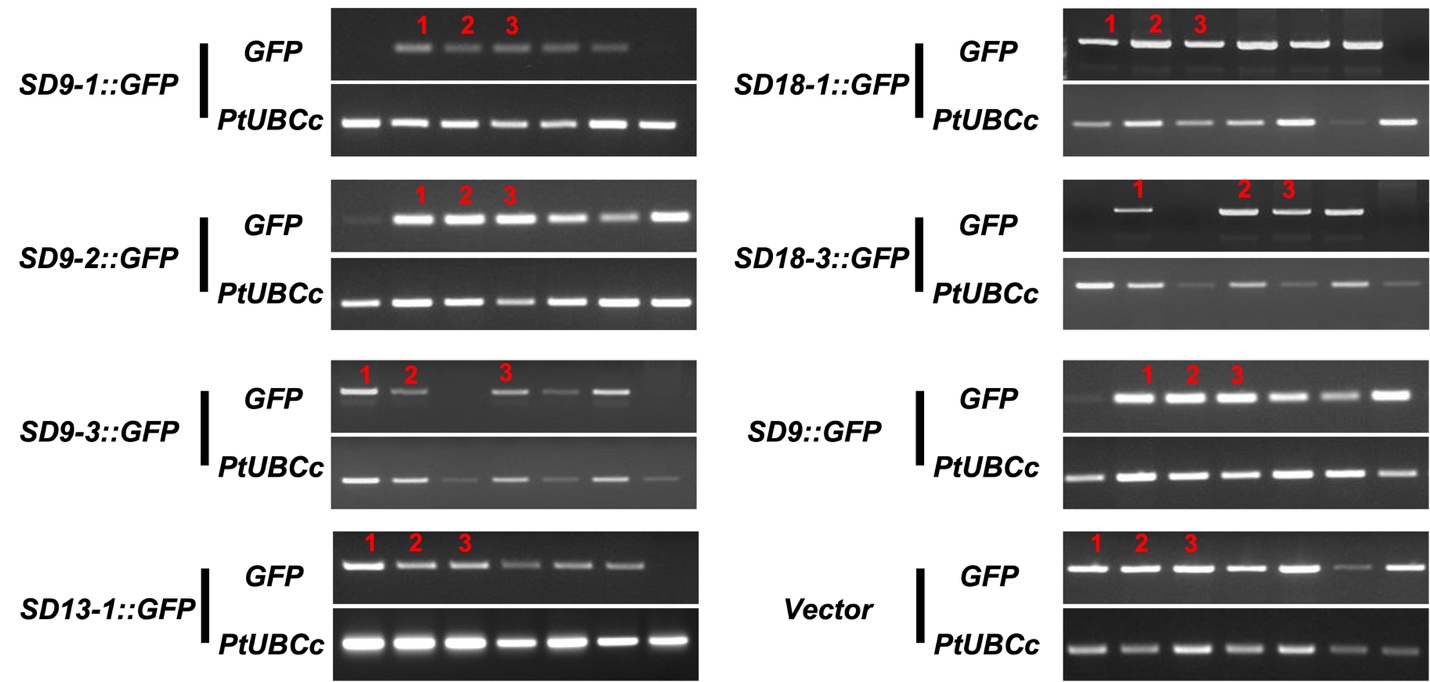


Figure S2. **Genotyping of transgenic poplar clones.**

Genomic DNA was extracted from fully expanded leaves from the original plants of each transgenic events before propagation. PtUBCc was used as a reference gene. Transgenic lines used in stress treatment experiments are indicated by numbers 1-3 for each promoter.


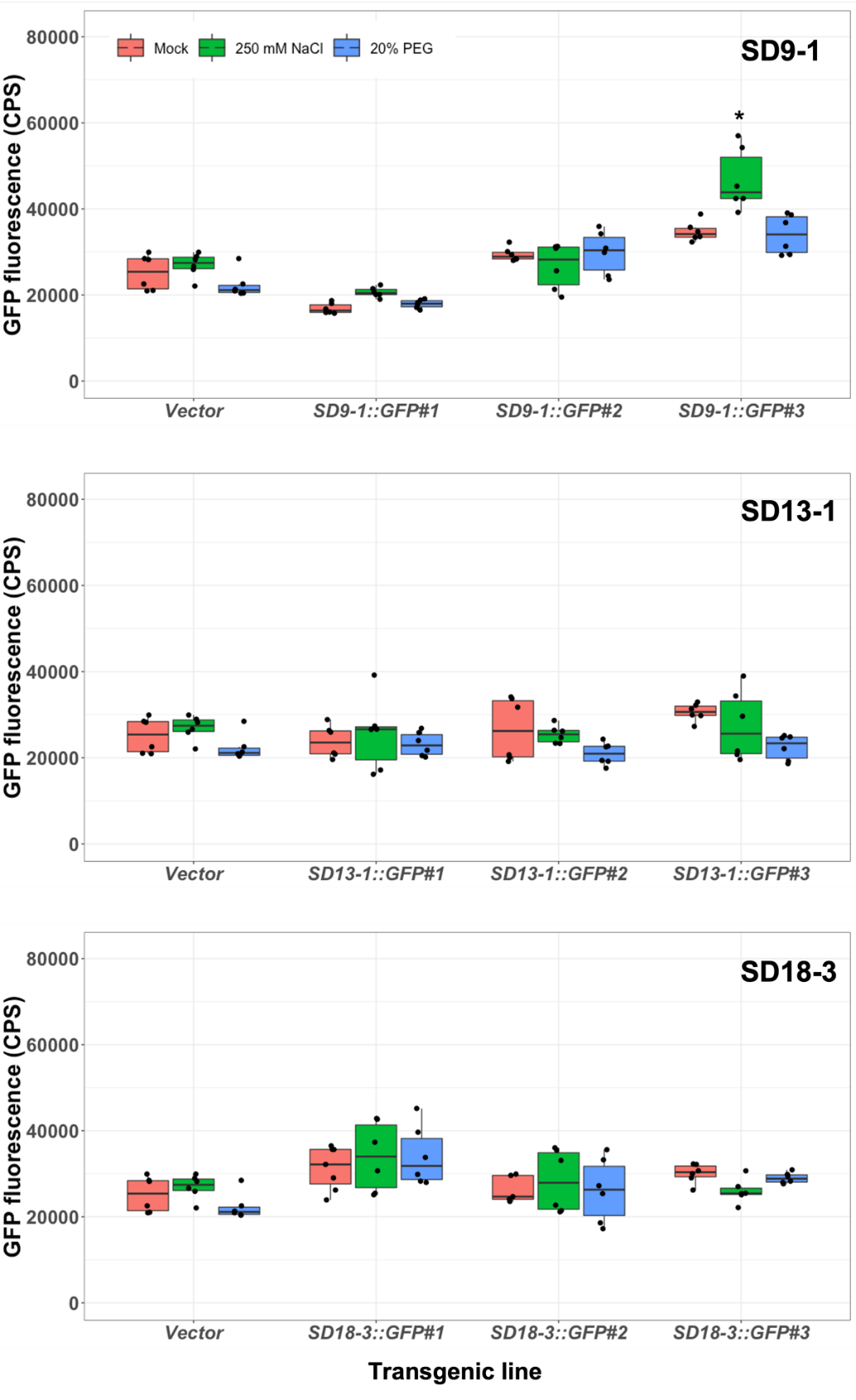


Figure S3. **GFP induction in stably transformed poplar harboring non-activated synthetic promoters.**

Table S1. **Oligonucleotide list**

| Oligonucleotide ID | Sequence | Used figure |
| --- | --- | --- |
| Full SD9 motif_for | ggagGTCGAGGTACGCGCAAGCTGGTCGAGGTACGCGCAAGCTGGTCGAGGTACGCGCAAGCTGGTCGAGGTACGCGCAAGCTGGTCGAGGTACGCGCAAGCTGGTCGAGGTACGCGCAAGCTGGTCGAGGTACGCGCAAGCTG | Figure 2 |
| Full SD9 motif_rev | agtaCAGCTTGCGCGTACCTCGACCAGCTTGCGCGTACCTCGACCAGCTTGCGCGTACCTCGACCAGCTTGCGCGTACCTCGACCAGCTTGCGCGTACCTCGACCAGCTTGCGCGTACCTCGACCAGCTTGCGCGTACCTCGAC | Figure 2 |
| PtUBCc_for | CTGAAGAAGGAGATGACAGCACCA | Figure S2 |
| PtUBCc_rev | GCATCCCTTCAACACAGTTTCACG | Figure S2 |
| GFP_for | GGATCTGGATCTGAGTCTGATGAGTCT | Figure S2 |
| GFP_rev | CCTTGAAATCTCCGATCACTCTTCCAG | Figure S2 |
